# The impact of insulin pump therapy compared to multiple daily injections on complications and mortality in type 1 diabetes: A real‐world retrospective cohort study

**DOI:** 10.1111/dom.16455

**Published:** 2025-05-19

**Authors:** Sophie Haughton, David Riley, Simon Berry, Muhammad Fahad Arshad, Aikaterini Eleftheriadou, Matthew Anson, Yew Wen Yap, Daniel J. Cuthbertson, Rayaz A. Malik, Shazli Azmi, Uazman Alam, Ahmed Iqbal

**Affiliations:** ^1^ Sheffield Teaching Hospitals Sheffield UK; ^2^ Diabetes & Endocrinology Research Institute of Life Course and Medical Sciences, University of Liverpool and Liverpool University Hospitals NHS Foundation Trust Liverpool UK; ^3^ School of Medicine and Population Health The University of Sheffield Sheffield UK; ^4^ Division of Diabetes, Endocrinology and Gastroenterology Faculty of Biology, Medicine and Health, University of Manchester Manchester UK; ^5^ Weill Cornell Medicine Doha Qatar

**Keywords:** cohort study, diabetes complications, insulin pump therapy, real‐world evidence, type 1 diabetes

## Abstract

**Aims:**

Clinical trials have demonstrated the benefits of insulin pump therapy compared with multiple daily injections (MDI) in type 1 diabetes. However, contemporaneous real‐world data are limited. This study investigated the real‐world impact of insulin pump therapy compared with MDI.

**Materials and Methods:**

A retrospective cohort study of adults with type 1 diabetes was performed on the TriNetX platform, a global network providing access to anonymised medical records. Outcomes analysed include HbA1c, diabetic ketoacidosis, macro‐ and microvascular complications and all‐cause mortality. The five‐year follow‐up period, between January 2018 and March 2025, was divided into time windows for analysis.

**Results:**

95 122 individuals with type 1 diabetes were identified. After propensity score matching for confounders including age, ethnicity, gender, chronic kidney disease, retinopathy, HbA1c and microalbuminuria, 17 124 patients remained in both the pump and MDI cohorts. The absolute reduction in HbA1c was comparable at five years (−5.3 mmol/mol [−0.5%] in the pump group and −4.5 mmol/mol [−0.4%] in MDI). Overall mortality was lower (RR = 0.716 [95% CI 0.639–0.803], *p* < 0.001) in those on a pump compared to MDI. The occurrence of diabetic ketoacidosis was lower in the pump group compared to MDI (RR = 0.848 [95% CI 0.786–0.915], *p* < 0.001). The risk of diabetic retinopathy was increased in the pump group (RR = 1.331 [95% CI 1.247–1.420], *p* < 0.001).

**Conclusions:**

Insulin pump therapy was associated with lower all‐cause mortality and risk of diabetic ketoacidosis, but an increased risk of diabetic retinopathy compared with MDI. This result should be interpreted with caution due to potential differences in retinal screening frequency and subsequent bias.

## 1 INTRODUCTION

Type 1 diabetes is a major cause of morbidity and premature mortality. It affects over 8 million people worldwide, and prevalence is expected to rise to between 13.5 and 17.4 million by 2040.^1^ It is characterised by autoimmune beta cell destruction causing insulin deficiency and hyperglycaemia, which requires life‐long administration of exogenous insulin.^2^ Hyperglycaemia predisposes to a range of serious and costly complications, including diabetic eye, foot and kidney disease, coronary artery disease and cerebrovascular disease. In 2017, $237 billion was spent on direct costs in the US relating to diabetes care.^3^ The Diabetes Control and Complications Trial (DCCT) and the Epidemiology of Diabetes Interventions and Complications (EDIC) demonstrated that intensive glycaemic control reduced the risk of macro‐ and microvascular complications.^4^

Insulin pumps, or continuous subcutaneous insulin infusion, are an important therapeutic option for people with type 1 diabetes.^5^ Insulin pump therapy more closely mimics physiological glucose homeostasis by continuously infusing rapid‐acting insulin analogues, compared to multiple daily injections (MDI) that typically consist of longer acting basal insulin and shorter acting boluses.^6^ Insulin pump use in the US between 2016 and 2018 was estimated at ~63% of adults with type 1 diabetes, whereas it was only ~11% in the UK between 2021 and 2022.^7^, ^8^, ^9^, ^10^

Older meta‐analyses (2012) demonstrated that overall, insulin pumps and MDI had comparable outcomes in relation to glycaemic control and hypoglycaemia.^11^ However, a 2021 meta‐analysis of adults with type 1 diabetes showed that insulin pumps were superior to MDI with regard to HbA1c and glucose variability, without increasing severe hypoglycaemic episodes, but were associated with an increase in diabetic ketoacidosis (DKA).^12^ Improvements in HbA1c with insulin pump therapy are less marked when MDI regimens use rapid‐acting analogues, rather than human insulin.^13^ In a study by Azmi et al., insulin pump therapy was associated with an improvement in small nerve fibre morphology, despite comparable glycaemic control in patients on MDI.^14^ Another large prospective study, primarily in adolescents with type 1 diabetes, also demonstrated lower rates of peripheral nerve abnormalities and retinopathy in those on insulin pump therapy.^15^ Data from the Swedish National Diabetes Registry (2005–2012) have shown that insulin pump use is associated with lower cardiovascular mortality.^16^

Randomised controlled trials of insulin pump therapy are prone to participant selection bias, have a relatively short duration of follow‐up, and there are substantial differences in the intensity of device education and participant supervision. This large, real‐world study aimed to evaluate glycaemic control, incidence of diabetes‐related complications and mortality, reflecting more contemporary technology and MDI regimens (since 2018), in individuals using pump therapy compared to MDI in routine practice.

## 2 METHODS

### 2.1 Network characteristics

A retrospective cohort study was performed using the TriNetX platform. TriNetX is a global collaborative network, which provides access to real‐time electronic medical records (diagnoses, procedures, medications and laboratory values) from more than 110 million patients across 141 healthcare organisations, primarily in North America and Western Europe. Data were collected from secondary care institutions. The database was interrogated on 20th March 2025. Details of the TriNetX platform have been described previously.^17^

### 2.2 Building cohorts in TriNetX

ICD‐10‐CM, the National Library of Medicine, TriNetX and SNOMED codes were used to identify inclusion and exclusion criteria, and the presence or absence of relevant outcomes. The inclusion and exclusion criteria and the specific codes used can be found in the Supporting Information. Analysis was of data from people aged 18 years or older with a diagnosis of type 1 diabetes. Patients were divided into two cohorts: insulin pump users and MDI users.

The index event, at which the time window begins, was defined as a code entry of type 1 diabetes, which had to occur on or after 1st January 2018. For patients in the insulin pump cohort, the presence of an insulin pump must be recorded within a year of diagnosis. We used intention‐to‐treat analysis and assumed that once coded, a pump was used throughout the entire period.

Propensity score matching (PSM) is performed within the TriNetX platform. We used PSM to reduce confounding and simulate the conditions of a randomised comparison within our observational dataset. A logistic regression model was used to estimate each participant's probability (propensity score) of receiving the intervention based on baseline characteristics. Individuals in the pump group were then matched to those in the MDI group with similar propensity scores using nearest‐neighbour matching without replacement. This approach aimed to balance key covariates between groups, allowing for a more robust estimation of the association between the intervention and outcomes. The technical summary of our PSM is found in the Supporting Information. The groups were matched 1:1 for gender, ethnicity (% White ethnicity), age, chronic kidney disease (CKD), retinopathy, HbA1c and microalbuminuria. The groups were considered well‐matched if the standardised mean difference (SMD) is <0.1.

To avoid the potential inclusion of individuals with type 2 diabetes, patients were excluded if there had been any prior oral hypoglycaemic agent use. Individuals were excluded if they did not have an encounter with a healthcare provider at least 1 year after coding for type 1 diabetes.

### 2.3 Outcomes

The analysis was based on the following outcomes: mortality, HbA1c, DKA, diabetic foot ulcers, diabetic retinopathy, ischaemic heart disease (IHD), acute myocardial infarction (MI) and cerebral infarction and/or transient ischaemic attack (TIA). Data on continuous glucose monitor (CGM) use and number of clinic visits are included to illustrate potential between‐group differences. The first time an outcome was recorded within the time window was counted in the analysis, except for HbA1c, in which the latest laboratory result was recorded.

Statistical analysis was performed within the TriNetX platform. Normally distributed baseline characteristics are presented as mean and standard deviation. Risk ratio (risk for insulin pump cohort/risk for MDI cohort) and 95% CIs are presented for two time windows: 1–2 and 2–5 years (Figure S1). T‐Test statistics testing for the difference between the cohorts was performed and *p* < 0.05 was deemed statistically significant. HbA1c values were collected in yearly time windows, analysed as % and converted to mmol/mol for publication. The 0–1‐year time window is excluded from the main analysis due to uncertainty around the time of pump initiation within the first year and is retained in the Supporting Information. The end point for the follow‐up period is 5 years after diagnosis, unless this occurred within 5 years of our database search, or if data is no longer available for the patient. In these instances, the patient is subsequently censored.

*E*‐values are calculated for statistically significant risk ratios to determine the minimum strength of association that an unmeasured confounder would require with both the exposure and outcome, conditional on the measured covariates, to fully explain away a specific exposure–outcome association. A high *E*‐value implies that significant unmeasured confounding variables would be needed to nullify the effect estimate, whereas a low *E*‐value indicates that only modest unmeasured confounding variables would suffice for this purpose.^18^ It was not possible to calculate *E*‐values for HbA1c, CGM use or visits due to the character of the data. Where a result is not significant, there will be no *E*‐value.

## 3 RESULTS

### 3.1 Baseline characteristics

A search of the database identified 95 122 people with type 1 diabetes matching the inclusion criteria, including 17 260 using an insulin pump (Table S4). Those using an insulin pump were more likely to be younger, white, and female. Approximately 85% of this data is derived from the US (98% of pump data and 82% of MDI) (Table S5). After PSM, 17 124 patients with type 1 diabetes were included in each group (Table 1). The two cohorts were well matched for age, ethnicity, gender, CKD, retinopathy and microalbuminuria. However, the SMD for HbA1c was 0.183, indicating that the groups were not well matched: HbA1c was lower in the insulin pump group (HbA1c 67.2 mmol/mol ± 20.8 or 8.3% ± 1.9%) compared to the MDI group (71.6 mmol/mol ± 26.2 or 8.7% ± 2.4%). Baseline CGM use was 12.9% in the insulin pump group and 4.6% in the MDI group (*p* < 0.001).

**TABLE 1:** Baseline characteristics after propensity score matching (PSM).

Post PSM	Pump	MDI	SMD
Totals	17 124	17 124	‐
Mean age ± SD	36.2 ± 19.6	36.2 ± 19.6	<0.001
White %	84.3%	84.2%	0.002
Female %	52.3%	52.7%	0.009
Chronic kidney disease %	10.8%	10.7%	0.004
Retinopathy %	13.0%	12.6%	0.010
Mean HbA1c (mmol/mol ± SD) [% ± SD]	67.2 ± 20.8 [8.3 ± 1.9]	71.6 ± 26.2 [8.7 ± 2.4]	0.183
Microalbuminuria (mg/Dl)	22.0 ± 259.8	12.6 ± 104.5	0.048

Abbreviations: SD, standard deviation; SMD, standardized mean difference.

### 3.2 Mortality

**FIGURE 1:**
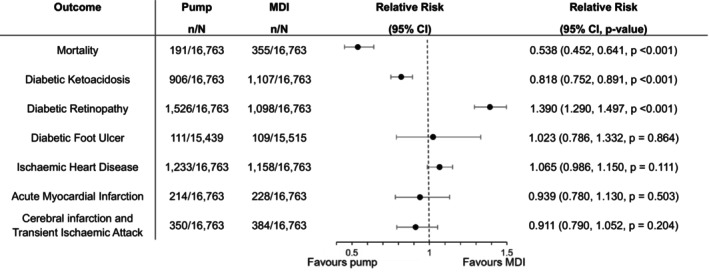
Outcomes from time window 1–2 years. *N*, total number in cohort; *n*, number in group with the outcome.

**FIGURE 2:**
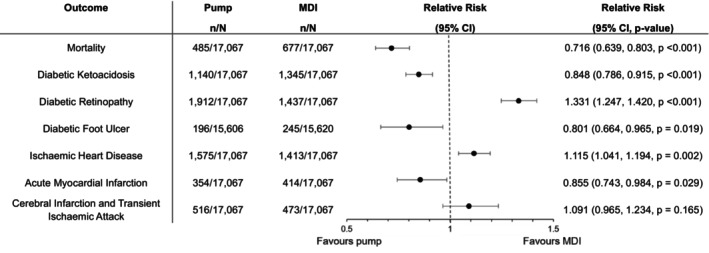
Outcomes from time window 2–5 years. *N*, total number in cohort; *n*, number in group with the outcome.

There were relative risk reductions in the pump group compared to the MDI group in mortality across both time periods (*p* < 0.001) (Figures 1 and 2).

### 3.3 Glycaemic control

#### 3.3.1 HbA1c

**FIGURE 3:**
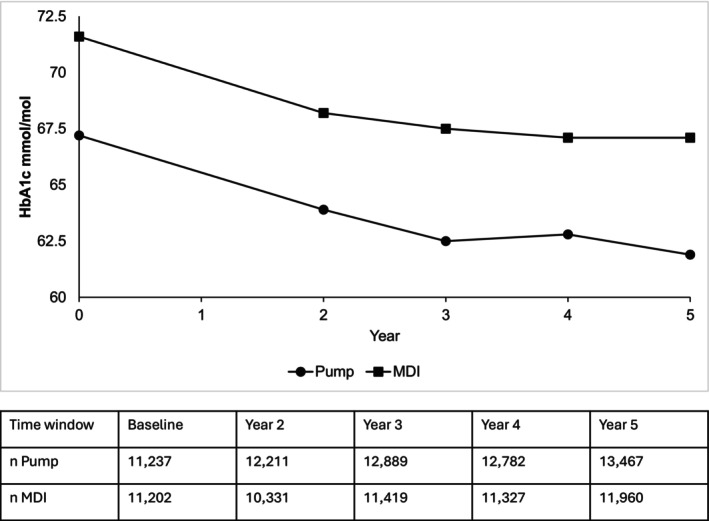
Mean HbA1c at the end of each time window with insulin pump (circles) and MDI (squares) and table of number (*n*) in the group with a HbA1c outcome.

An absolute reduction in HbA1c of 5.3 mmol/mol (0.5%) and 4.5 mmol/mol (0.4%) with insulin pump and MDI, respectively, was observed comparing baseline to year 5 (Table S6). Yearly mean HbA1c data, alongside the number of patients with HbA1c data available, are shown in Figure 3. After 5 years, the mean HbA1c in the insulin pump group was lower at 61.9 mmol/mol or 7.8% (±18.5 mmol/mol or 1.7%) versus 67.1 mmol/mol or 8.3% (±23.1 mmol/mol or 2.1%) in the MDI group (*p* < 0.001).

#### 3.3.2 CGM usage

The proportion of patients using a CGM in the insulin pump group was higher than the MDI at 5 years (35.7% vs. 17.9%; *p* < 0.001).

#### 3.3.3 DKA

The insulin pump group had a lower incidence of DKA compared to the MDI group across both time periods (*p* < 0.001) (Figures 1 and 2).

### 3.4 Microvascular complications

#### 3.4.1 Diabetic foot ulcers

From years 2–5, there was a lower incidence of diabetic foot ulcers in insulin pump users compared to MDI (*p* = 0.019). No difference was observed in years 1–2 (Figures 1 and 2).

#### 3.4.2 Diabetic retinopathy

The incidence of diabetic eye disease was higher in the insulin pump group across both time periods (*p* < 0.001) (Figures 1 and 2).

### 3.5 Macrovascular complications

#### 3.5.1 IHD

There was an excess incidence of IHD in years 2–5 in the insulin pump group (*p* = 0.002), and no difference in years 1–2 (Figures 1 and 2).

#### 3.5.2 MI

There was a lower risk of MI in years 2–5 in the insulin pump group (*p* = 0.029), and no difference in years 1–2 (Figures 1 and 2).

#### 3.5.3 Cerebral infarction and TIA

There was no difference in the incidence of cerebral infarction and TIA in either period (Figures 1 and 2).

### 3.6 Number of clinic visits

Across the five‐year window, there were more clinic visits (*p* < 0.001) in the insulin pump group (mean *n* = 56.8) compared to the MDI group (*n* = 51.5).

### 3.7 *E*‐value

*E*‐values for the outcomes are presented in the Supporting Information. *E*‐values for mortality, DKA, diabetic foot ulcers, diabetic retinopathy and MI were greater than 1.5. That is, an unmeasured confounder would need to have a minimum strength of association with both the exposure and the outcome of greater than 1.5 to fully explain away the treatment–outcome association; we believe such a confounder is unlikely. The *E*‐value for IHD at 2–5 years was 1.47, which suggests that the observed association of a greater incidence of IHD in the insulin pump group may be influenced by unmeasured confounding, and as such, should be interpreted with caution.

## 4 DISCUSSION

We investigated the real‐world impact of pump therapy compared to MDI on complications and mortality in type 1 diabetes. In a cohort of 34 248 individuals with type 1 diabetes, the use of insulin pumps was associated with a significant and clinically meaningful reduction in all‐cause mortality over five years, compared to MDI. Both the insulin pump and MDI groups exhibited clinically important absolute reductions in HbA1c over the five‐year period, amounting to 5.3 mmol/mol (0.5%) and 4.5 mmol/mol (0.4%), respectively. Additionally, there was a reduced risk of DKA and diabetic foot ulcers from years 2 to 5 in the insulin pump group.

Our study also demonstrates an increased relative risk of diabetic retinopathy for insulin pump users compared to MDI. These findings contrast with a longitudinal study of adolescents from Australia where insulin pump therapy reduced rates of retinopathy compared to MDI.^15^ In our study, the patients were older and so the populations are not directly comparable. A systematic review and meta‐analysis including 24 studies with 9302 patients found a relative risk of incident diabetic retinopathy of 0.45 with insulin pump therapy compared to MDI.^19^ A more recent Scottish retrospective cohort study found a smaller proportion of adults had progression of diabetic retinopathy with insulin pumps compared to MDI therapy over 2.3 years of follow‐up.^20^ In a cross‐sectional study from two large centres in the US, Ferm and colleagues also showed that insulin pump use in type 1 diabetes was independently associated with a lower likelihood of diabetic retinopathy.^21^ However, a recent Danish study showed no difference in the overall short‐ and long‐term risk of diabetic retinopathy worsening, or ocular intervention compared to MDI treatment.^22^ One putative mechanistic explanation for the increased rates of diabetic retinopathy in our study may be that insulin pump commencement resulted in a more rapid improvement in glycaemic control. Early worsening of diabetic retinopathy after rapid improvement in glycaemia is a well‐known phenomenon.^23^, ^24^ While the absolute reduction in HbA1c is comparable between the insulin pump and MDI groups at year 5, we did not capture indices of glycaemic variability, which include fluctuations in day‐to‐day glucose such as glucose flux in recovery from hypoglycaemia and post‐prandial rises.^25^ There is evidence that early damage to neuroretinal cells is linked to glycaemic variability.^26^ In the DCCT study, those in the intensive therapy arm exhibited early worsening of diabetic retinopathy in the first two years, but had sustained and substantial retinal improvement subsequently.^27^ Post‐hoc analyses of DCCT data suggest that within‐day glycaemic variability may not play an important role in the development of microvascular complications beyond the impact of mean glucose.^28^ However, a key limitation of these analyses is that glycaemic variability was derived from 7‐point self‐monitoring capillary blood glucose, which does not capture the granularity of variability compared to CGM. Another potential explanation for the elevated rates of retinopathy among insulin pump users may be a greater burden of hypoglycaemia, aggravating inflammation.^29^, ^30^, ^31^ A key factor for considering pump therapy is managing problematic hypoglycaemia. While we could not directly measure hypoglycaemia burden in our study, the lower baseline HbA1c in the pump group may suggest higher exposure to hypoglycaemic events. Overall, our observation of an increased risk of retinopathy with pump use should be interpreted with caution, for the following reasons: (1) our real‐world study relies on routine reporting, thus our results may be confounded by a higher rate of diabetic retinopathy screening in the pump group; and (2) our results do not distinguish between the severity or progression of diabetic retinopathy, but rather its presence or absence. Information on the extent of eye disease through grading and screening rates could help to elucidate a more accurate and granular picture of diabetic eye disease.

Previous meta‐analyses have demonstrated an improvement in glycaemic control with insulin pump therapy, although reduced benefit has been found when comparing pumps to MDI regimens using newer insulin analogues.^12^, ^13^ Our data demonstrate a clinically meaningful improvement in glycaemia in both the insulin pump and the MDI groups. The mean HbA1c in the pump group was lower at 5 years but these results are difficult to interpret given the higher baseline HbA1c in the MDI group despite PSM. Although pump users were more likely to use a CGM than those on MDI, HbA1c fell by similar absolute amounts. The role of CGM in glycaemic control may be more significant than the insulin delivery method, as supported by a systematic review that reported similar glycaemic outcomes when comparing real‐time CGM use in conjunction with both insulin pumps and MDI.^32^ The CGM use data in this study likely underestimates the true prevalence of CGM use in this population, given that recent studies on CGM report uptake of almost 50% in patients with type 1 diabetes,^33^, ^34^ compared with 35.7% of pump users and 17.9% of MDI users in our study. We suspect that the missing CGM data is related to incomplete coding in the database and as such should be interpreted cautiously.

Our findings of a reduction in all‐cause mortality with insulin pump use are consistent with results from the Swedish National Diabetes Register involving 2441 patients, which showed reduced all‐cause mortality in pump users compared to MDI (HR = 0.73 [95% CI 0.58–0.92], *p* = 0.007).^16^ The precise mechanisms of improvement in all‐cause mortality in our study remain unclear. The Swedish cohort study found improvements in fatal coronary heart disease and fatal cardiovascular disease in pump users, starting from the first year of treatment.^16^ However, when cumulative fatal and non‐fatal cases were considered, there was no reduction in coronary heart disease or cardiovascular disease. Our results show no difference in the incidence of IHD or MI in years 1–2. Between years 2 and 5, pump use is associated with increased IHD risk yet decreased MI risk. Notably, the *E*‐value for this IHD result is 1.47 meaning that it may be subject to significant unmeasured cofounding which could explain away the observed association. Longer‐term follow‐up may elucidate this seemingly contradictory association between pump use and IHD versus MI.

There was a reduced risk of DKA in both time windows, which contrasts with previous studies showing increased DKA in pump users,^6^ although a 2009 review found no difference in DKA rates between pumps and MDI.^35^ In a randomised controlled trial comparing pumps (*n* = 119) with MDI (*n* = 116) over 2 years, the number of DKA episodes at year 1 was higher in the pump group (17 vs. 5), but there was no difference at year 2.^36^ A relative risk reduction in diabetic foot ulcers was seen in the pump group compared to the MDI group in the 2‐to‐5‐year time window. Our results are consistent with previous studies showing improved measures of neuropathy in patients on insulin pump therapy which of course is a key driver of diabetic foot ulceration.^15^

### 4.1 Strengths

Our study had a large sample size. We only included data from 2018, ensuring that contemporary insulin pumps were compared with modern MDI regimens. The range of complications evaluated provides data on outcomes seldom reported in the literature and clinical trials, such as mortality and foot ulceration. We observed seemingly contradictory results relating to IHD and MI in the 2‐to‐5‐year window. There is a known lag in the development of macrovascular complications in type 1 diabetes, therefore evaluation of these treatment groups over a longer follow‐up period may reveal a clearer picture of the impact on cardiovascular disease, as it did in the DCCT and EDIC study.^37^ However, we considered the observed incidences of the outcomes in the 2‐to‐5‐year time window to be representative of the true effect. A delay from pump initiation minimises survival bias by evaluating the patients at least two years into their therapy, who should therefore be established on it. The first‐year results were excluded due to the potential for bias to be introduced by coding around the time of diagnosis.

### 4.2 Limitations

Despite PSM, HbA1c levels were higher in the MDI group compared to pump users. This could result in a higher risk of diabetes‐related complications in the MDI group, due to the legacy effect of hyperglycaemia. This may weaken the observed associations between MDI and negative outcomes. The proportion of patients using CGMs was higher in the pump group, which may further confound these associations.^32^ Laboratory value measurements were not available for all individuals, due to incomplete coding or unavailable data, and so may not reflect the entire cohort. Data on the incidence of hypoglycaemia and severe hypoglycaemia would have also been useful.^38^ However, analysing hypoglycaemia data from this dataset would have been vulnerable to confounding and inconsistent retrospective reporting, introducing bias.

Hybrid closed loop pump systems have been shown to be superior to open loop systems with respect to glycaemic control.^39^, ^40^ A subgroup analysis for pump type would be helpful however, this was not possible as the TriNetX database is unable to distinguish between pump types. Diabetes duration is well known to increase the risk of adverse outcomes.^41^, ^42^ Although we did not directly evaluate disease duration, by using CKD, retinopathy and microalbuminuria in our PSM criteria, we effectively matched patients for their microvascular disease burden, which we consider a proxy for disease duration. We acknowledge that disease duration may determine hyperglycaemia exposure above and beyond this. While we matched for age, gender and ethnicity, other important demographic variables may have impacted our results, especially socioeconomic factors like household income and parental education, given that pump users are more likely to be socioeconomically priviledged.^43^, ^44^, ^45^ Noting that a greater proportion of the data was derived from the US for the pump group compared to MDI, there are likely intercountry factors, such as healthcare access and health education, which were not captured in our matching criteria. We used *E*‐values to help quantify how strong any such unmeasured cofounders would have to be to explain away the observed associations. Only one of the outcomes demonstrated an *E*‐value of <1.5 (IHD in years 2–5) as has been discussed above, and as such, we find that the impact of any such cofounders on the rest of the results is unlikely.

## 5 CONCLUSION

This large, real‐world study has provided a realistic understanding of insulin pump use outside the close monitoring that occurs within clinical trials. In our cohort of patients with type 1 diabetes over five years, insulin pump use was associated with lower all‐cause mortality and risk of DKA. The risk of diabetic retinopathy was higher in pump users, however, this result should be interpreted with caution due to possible differences in screening frequency. Clinically meaningful reductions in HbA1c were seen in both the insulin pump and MDI groups.

## AUTHOR CONTRIBUTIONS

S.H. was involved in conceptualisation, formal analysis and wrote the first draft of the manuscript. D.R. contributed to conceptualisation, data curation, methodology and formal analysis. S.B. undertook formal analysis and writing. M.F.A. contributed to conceptualisation and methodology. A.E. performed formal analysis and contributed to the writing of the manuscript. R.A.M., D.J.C., M.A. and Y.W.Y. were involved in conceptualisation and methodology. S.A. contributed to conceptualisation and methodology. U.A. was responsible for conceptualisation, methodology and oversight of the analyses. A.I. contributed to conceptualisation, methodology and formal analysis. All authors provided critical input on multiple versions of the manuscript. All authors approved the final submitted version. U.A. and A.I. are the guarantors of this work, and as such, had full access to all of the data in the study and take full responsibility for data integrity and accuracy.

## FUNDING INFORMATION

This research received no specific grant from any funding in the public, commercial, or not‐for‐profit sectors.

## CONFLICT OF INTEREST STATEMENT

U.A. has received honoraria from Viatris, Grünenthal, Eli Lilly, Sanofi and Procter & Gamble for educational meetings, investigator‐led funding from Proctor & Gamble and received sponsorship to attend educational meetings from Daiichi Sankyo and Sanofi. A.I. has received research support from Abbott and Dexcom Inc. plus speaker fees from AstraZeneca, Boehringer Ingelheim, and Eli Lilly. S. B. has received research support from Dexcom and Tandem Diabetes Care. R.A.M. has received honoraria from Novo Nordisk, Viatris, Eli Lilly, Sanofi and Procter & Gamble for educational meetings and has received investigator‐led funding from Novo Nordisk and Procter & Gamble. D.J.C. has received investigator‐initiated grants from AstraZeneca and Novo Nordisk, and support for education from Perspec‐tum. M.A. receives a fellowship from the Novo Nordisk UK research foundation and JDRF. Y.W.Y., D.R., S.H., A.E. and M.F.A. have no competing interests or disclosures.

## PEER REVIEW

he peer review history for this article is available at https://www.webofscience.com/api/gateway/wos/peer-review/10.1111/dom.16455.

## Supporting information


**Data S1.** Supporting information.

**Data S2.** Supporting information.

**Data S3.** Supporting information.

## Data Availability

To gain access to the data in the TriNetX research network, a request can be made to TriNetX (https://live.trinetx.com), but costs may be incurred, a data sharing agreement would be necessary and no patient identifiable information can be obtained. No data from Liverpool University NHS Foundation Trust or Sheffield Teaching Hospitals NHS Foundation Trust were utilised in this analysis.
