# Supplementary figures and images for: The impact of insulin pump therapy compared to multiple daily injections on complications and mortality in type 1 diabetes: A real‐world retrospective cohort study
Source: Diabetes Obes Metab. 2025 May 19;27(8):4239–47. doi: 10.1111/dom.16455 (PMC12232336; doi:10.1111/dom.16455)

## Slide 1
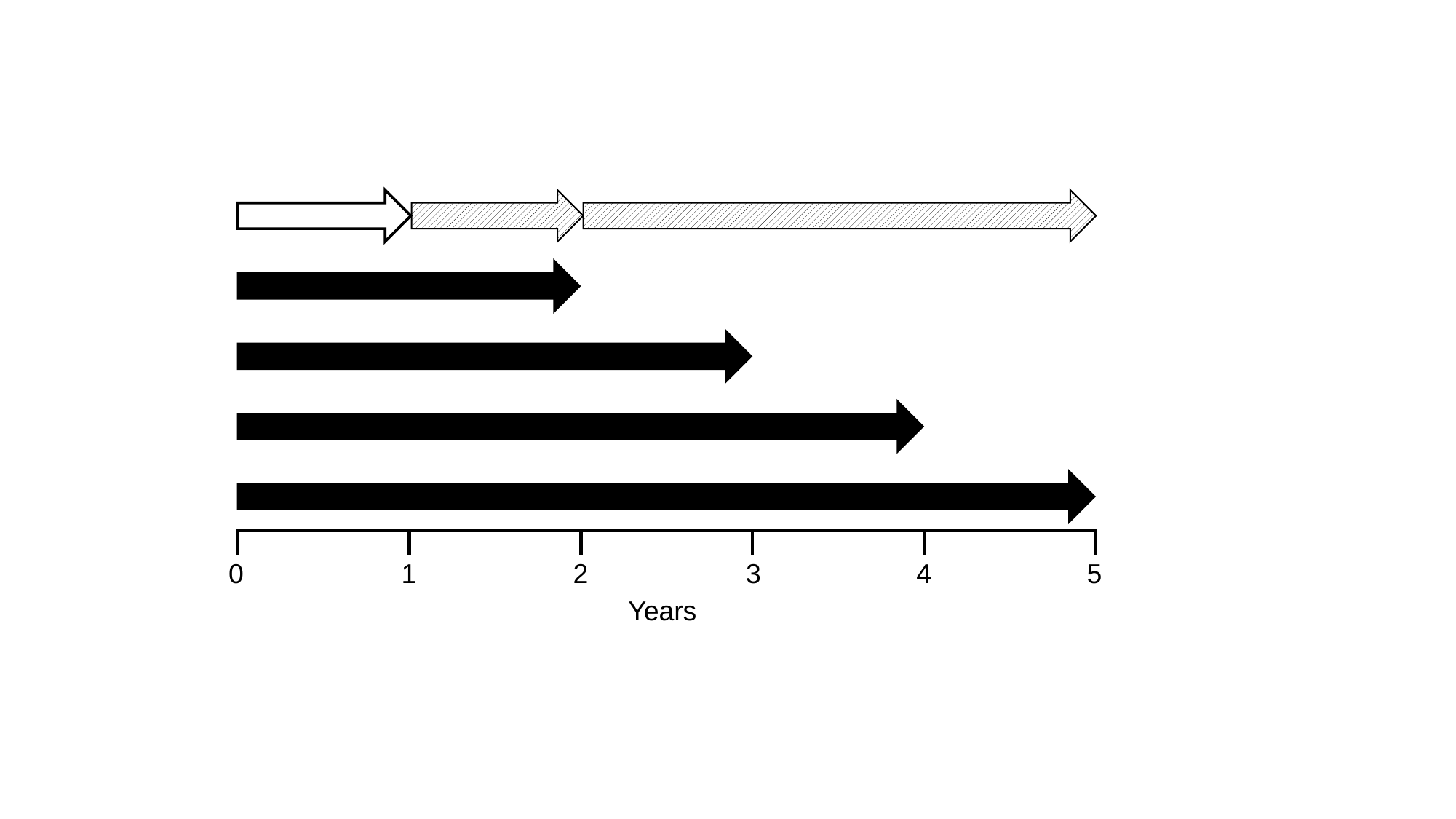

0
1
2
3
4
5
Years

Supplement: Supplementary file 1 — Data S1. Supporting information. [file DOM-27-4239-s003.pptx]
