# Supplementary material for: The impact of insulin pump therapy compared to multiple daily injections on complications and mortality in type 1 diabetes: A real‐world retrospective cohort study
Source: Diabetes Obes Metab. 2025 May 19;27(8):4239–47. doi: 10.1111/dom.16455 (PMC12232336; doi:10.1111/dom.16455)
Supplement: Supplementary file 2 — Data S2. Supporting information. [file DOM-27-4239-s002.docx]

Supplementary Methods

| *Inclusion Criteria* | *Code* | *Definition* |
| --- | --- | --- |
| Both cohorts |  |  |
| Must have | Age (at least 18 years (most recent occurrence)) |  |
| Must have | NLM:VA:HS501 | Medications, Insulin |
| Cannot have | NLM: VA: HS502 | Oral hypoglycemic agents,oral |
| Cannot have | NLM: VA: HS509 | Hypoglycemic agents,other |
| Must have | UMLS: ICD10CM:E10  occurred on or after Jan 1, 2018 | Type 1 diabetes mellitus |
| Must have | TNX:Visit  occurred at least 12 months after date of T1DM diagnosis |  |
| OR | Deceased |  |
| OR | UMLS: ICD10CM:R99 | Ill-defined and unknown cause of mortality |
| Pump cohort |  |  |
| Must have | UMLS: ICD10CM:Z96.41 | Presence of insulin pump (external) (internal) |
| OR | UMLS: SNOMED:180178009 | Continuous subcutaneous infusion of insulin |
| OR | UMLS: ICD10CM:Z46.81 | Encounter for fitting and adjustment of insulin pump |
| MDI cohort |  |  |
| Cannot have | UMLS: SNOMED:180178009 | Continuous subcutaneous infusion of insulin |
| OR | UMLS: ICD10CM:Z96.41 | Presence of insulin pump (external) (internal) |
| OR | UMLS: ICD10CM:Z46.81 | Encounter for fitting and adjustment of insulin pump |
| Characteristics for Propensity Score Matching |  |  |
|  | AI | Age at Index |
|  | 2106-3 | White |
|  | F | Female |
|  | M | Male |
|  | N18 | Chronic kidney disease (CKD) |
|  | 9037 | Hemoglobin A1c/Hemoglobin.total in Blood |
|  | E10.3 | Type 1 diabetes mellitus with ophthalmic complications |
|  | 9037 | Microalbumin [Mass/volume] in Urine |
| Outcomes |  |  |
| Diabetic Foot Ulcer | UMLS: ICD10CM:E10.621  UMLS: ICD10CM:Z86.31  UMLS: ICD10CM:L97.5  UMLS: ICD10CM:L97.4 | Type 1 diabetes mellitus with foot ulcer  Personal history of diabetic foot ulcer  Non-pressure chronic ulcer of other part of foot  Non-pressure chronic ulcer of heel and midfoot |
| Mortality | Deceased  UMLS: ICD10CM:R99 | Deceased  Ill-defined and unknown cause of mortality |
| Diabetic Retinopathy | UMLS: ICD10CM:E10.31  UMLS: ICD10CM:E10.35  UMLS: ICD10CM:E10.33  UMLS: ICD10CM:E10.34  UMLS: ICD10CM:E10.32 | Type 1 diabetes mellitus with unspecified diabetic retinopathy  Type 1 diabetes mellitus with proliferative diabetic retinopathy  Type 1 diabetes mellitus with moderate nonproliferative diabetic retinopathy  Type 1 diabetes mellitus with severe nonproliferative diabetic retinopathy  Type 1 diabetes mellitus with mild nonproliferative diabetic retinopathy |
| Ischaemic Heart Disease | UMLS: ICD10CM:I20-I25 | Ischemic heart diseases |
| Acute Myocardial Infarction | UMLS: ICD10CM:I21 | Acute myocardial infarction |
| Cerebral Infarction and Transient Ischaemic Attack | UMLS: ICD10CM:I63  UMLS: ICD10CM:Z86.73 | Cerebral infarction  Personal history of transient ischemic attack (TIA)  cerebral infarction without residual deficits |
| Visits | TNX:Visit |  |
| Diabetic ketoacidosis | UMLS: ICD10CM:E10.1  UMLS: ICD10CM:E13.1 | Type 1 diabetes mellitus with ketoacidosis  Other specified diabetes mellitus with ketoacidosis |
| Haemoglobin A1c | TNX:9037 | Hemoglobin A1c/Hemoglobin.total in Blood |
| CGM initiated | UMLS:CPT:1  014304 | Ambulatory continuous glucose monitoring of interstitial tissue fluid via a subcutaneous sensor for a minimum of 72 hours |

***Supplementary Table 2.*** Codes used for interrogation of database. Abbreviations: NLM – National Library of Medicine; UMLS – Unified Medical Language System (UMLS); ICD-10-CM - International Classification of Diseases, Tenth Revision, Clinical Modification; TNX – TriNetX
