# Supplementary material for: The impact of insulin pump therapy compared to multiple daily injections on complications and mortality in type 1 diabetes: A real‐world retrospective cohort study
Source: Diabetes Obes Metab. 2025 May 19;27(8):4239–47. doi: 10.1111/dom.16455 (PMC12232336; doi:10.1111/dom.16455)
Supplement: Supplementary file 3 — Data S3. Supporting information. [file DOM-27-4239-s001.docx]

**Supplementary Appendix**

Supplementary Appendix Table 1: Table of inclusion and exclusion criteria

| **Inclusion criteria** | | **Exclusion criteria** | |
| --- | --- | --- | --- |
| *Insulin pump* | *MDI* | *Insulin pump* | *MDI* |
| At least 18 years of age | | On oral or other hypoglycaemic agents | |
| Diagnostic coding of type 1 diabetes on or after 1^st^ January 2018 | | Living patients who did not have an encounter with a healthcare professional at least 1 year after coded diagnosis | |
| On insulin | |  | Presence of insulin pump or CSII |
| Presence of insulin pump or CSII within 1 year of type 1 diabetes code |  |  |  |

CSII *continuous subcutaneous insulin infusion*

Supplementary Appendix Table 2: Codes used for interrogation of database. Abbreviations: NLM – National Library of Medicine; UMLS – Unified Medical Language System (UMLS); ICD-10-CM - International Classification of Diseases, Tenth Revision, Clinical Modification; TNX – TriNetX

[See separate file]

Supplementary Appendix File 1: Technical summary of the propensity score matching

Propensity score matching is performed within the TriNetX platform. When balancing two cohorts a covariate matrix is created, with each column representing a different covariate and each row representing a patient. Logistic regression is performed for each patient with a value generated between 0 and 1; this predicts the probability of the patient being in the second cohort, cohort B. Logistic regression is used as the response variable is dichotomous, i.e. is the patient in cohort B, yes or no. Finally, for each patient in cohort A, the patient with the closest propensity score, who has not yet been matched, from cohort B is paired with them. This technique is referred to as “greedy nearest neighbour matching” and TriNetX uses a calliper of 0.1 pooled standard deviations when matching.

Supplementary Appendix Figure 1: Diagram of the time windows analysed, depicted with arrows – block arrows for HbA1c, hatched arrows for all non-laboratory clinical outcomes, blank arrow for excluded data

[See separate file]

Supplementary Appendix Table 3: Outcomes from time window 0 to 1 years. N = total number in cohort, n = number in group with the outcome

| **Outcome** | **Pump n/N** | **MDI n/N** | **RR (95% CI)** | **P value** |
| --- | --- | --- | --- | --- |
| Mortality | 170/17,124 | 276/17,124 | 0.616  (0.509, 0.745) | <0.001 |
| Diabetic Ketoacidosis | 1,641/17,124 | 1,586/17,124 | 1.035  (0.969, 1.105) | 0.309 |
| Diabetic Retinopathy | 1,982/17,124 | 1,251/17,124 | 1.584  (1.481, 1.695) | <0.001 |
| Diabetic Foot Ulcer | 250/16,022 | 255/16,064 | 0.983  (0.827, 1.169) | 0.846 |
| Ischaemic Heart Disease | 1,721/17,124 | 1,384/17,124 | 1.243  (1.162, 1.330) | <0.001 |
| Acute Myocardial Infarction | 396/17,124 | 330/17,124 | 1.200  (1.038, 1.387) | 0.013 |
| Cerebral Infarction and Transient Ischaemic Attack | 535/17,124 | 484/17,124 | 1.105  (0.979, 1.248) | 0.105 |

RR *risk ratio*

Supplementary Appendix Table 4: Baseline characteristics prior to propensity score matching (PSM)

| Pre PSM | Pump | MDI | SMD |
| --- | --- | --- | --- |
| Totals | 17,260 | 77,862 | - |
| Mean age ± SD | 36.1 ± 19.6 | 43.3 ± 20.8 | 0.357 |
| White % | 84.5% | 62.6% | 0.512 |
| Female % | 52.7% | 45.7% | 0.139 |
| Chronic Kidney Disease % | 10.7% | 17.7% | 0.201 |
| Retinopathy % | 13.0% | 10.2% | 0.090 |
| Mean HbA1c (mmol/mol ± SD) [% ± SD] | 67.2 ± 20.8  [8.3 ± 1.9] | 71.6 ± 27.3  [8.7 ± 2.5] | 0.157 |
| Microalbuminuria (mg/dL) | 21.9 ± 259.1 | 38.9 +/ 248.5 | 0.067 |

SD *standard deviation*; SMD *standardised mean difference*

Supplementary Appendix Table 5: Regional distribution of the dataset prior to propensity score matching

|  | **Pump (%)** | **MDI (%)** |
| --- | --- | --- |
| US | 98 | 82 |
| Elsewhere | <1 | 13 |
| Unknown | 1 | 5 |

Supplementary Appendix Table 6: Mean HbA1c rounded to one decimal place

|  | Baseline | Year 2 | Year 3 | Year 4 | Year 5 | Change baseline to year 5 |
| --- | --- | --- | --- | --- | --- | --- |
| Pump  mmol/mol ± SD  [% ± SD] | 67.2 ± 20.8  [8.3 ± 1.9] | 63.9 ± 18.7  [8.0 ± 1.7] | 62.5 ± 18.3  [7.9 ± 1.7] | 62.8 ± 18.7  [7.9 ± 1.7] | 61.9 ± 18.5  [7.8 ± 1.7] | -5.3  [-0.5] |
| MDI  mmol/mol [% ± SD] | 71.6 ±  26.2  [8.7 ± 2.4] | 68.2 ± 23.4  [8.4 ± 2.1] | 67.5 ± 23.2  [8.3 ± 2.1] | 67.1 ± 23.1  [8.3 ± 2.1] | 67.1 ± 23.1  [8.3 ± 2.1] | -4.5  [-0.4] |

SD *standard deviation*

Supplementary Appendix Table 7: E-values for the main outcomes in the insulin pump cohort compared to the MDI cohort

| **Time Window:** | 1-2 years | 2-5 years |
| --- | --- | --- |
| **Outcome:** | **E-Value (CI)** | **E-Value (CI)** |
| Mortality | 3.12 (2.49) | 2.14 (1.80) |
| Diabetic Ketoacidosis | 1.74 (1.49) | 1.64 (1.41) |
| Diabetic Foot Ulcer | N/A | 1.81 (1.23) |
| Diabetic Retinopathy | 2.13 (1.90) | 1.99 (1.80) |
| Ischaemic Heart Disease | N/A | 1.47 (1.25) |
| Acute Myocardial Infarction | N/A | 1.61 (1.14) |
| Cerebral Infarction and Transient Ischaemic Attack | N/A | N/A |

N/A *E-value not available i.e. when the association is insignificant*
